# Research on odor prediction methods for coal spontaneous combustion based on E-nose technology

**DOI:** 10.1038/s41598-025-30436-0

**Published:** 2025-12-02

**Authors:** Chen Shaojie, He Wentao, Li Dongming, Liang Shuaiheng, Qiao Tong, Duan Zuojin

**Affiliations:** College of Safety Science and Engineering, North China Institute of Science and Technology, Langfang, 065201 Hebei China

**Keywords:** E-nose, Odor prediction, CSC, PCA, SVM, Energy science and technology, Engineering

## Abstract

This study uses an electronic nose to predict Coal Spontaneous Combustion (CSC) based on odor information. In this study, we built a Programmable Temperature Coal Electronic Nose Testing Device (PTC E-nose). Its purpose was to detect odors from gases emitted by lignite during spontaneous combustion (30–200 °C). Odor characteristics at different stages of spontaneous combustion were analyzed, and principal component analysis (PCA) was used to illustrate their distribution across the various stages. Multiple machine learning models were employed to predict CSC stage. Results showed that acetaldehyde was the dominant volatile compound detected by the E-nose during the early stage of CSC, with a feature importance score of 0.38 in the random forest analysis. The response intensity of the acetaldehyde sensor showed a significant correlation with coal temperature (R²=0.97), suggesting its utility as an effective indicator for predicting spontaneous combustion, with benzene serving as an auxiliary predictor. The odor characteristics during different stages of CSC were distinctly different. PCA effectively differentiated among CSC stages (explaining 92.45% of the variance). The Principal Component Analysis-Support Vector Machine (PCA-SVM) model demonstrated the ability to identify characteristics of CSC odor and accurately classify stages, achieving recognition accuracy exceeding 95%. This study concludes that using E-nose technology for odor detection can be effectively used for monitoring and early warning of CSC. It is especially suitable for high-risk areas such as goaf areas, fractured coal seams, fault zones, and abandoned roadways.

## Introduction

Coal is a vital global energy resource, and its secure supply is essential for economic development and energy security. However, the coal production process is often accompanied by CSC disasters. CSC-induced fires can lead to significant casualties, substantial economic losses, and severe environmental pollution^1,2^. According to statistics, over 90% of coal seams in China are prone to spontaneous combustion, making CSC particularly prevalent during coal mining operations^3^. Despite extensive research, rapid and reliable early-warning techniques for CSC remain limited, underscoring the need for innovative predictive approaches to enhance safety in intelligent coal mines.

Among existing approaches for CSC prediction, gas analysis remains the most widely employed method^4–8^. It predicts the evolution of CSC by tracking changes in the concentration of characteristic gases (e.g., CO, C₂H₄, C₂H₂) generated during coal oxidation. However, this method can detect only a limited set of gaseous species and often fails to capture the complex odor signatures produced during combustion.

Recent studies have demonstrated that CSC is a multi-stage physicochemical process that emits numerous volatile compounds beyond conventional indicator gases, such as acetaldehyde, aromatic hydrocarbons, and ketones^9,10^. These odor compounds can reveal early combustion behavior. Yet, the detection techniques in these studies primarily rely on high-precision instruments that are expensive, time-consuming, and unsuitable for on-site monitoring. Consequently, there is a critical need for a cost-effective and rapid alternative capable of capturing the dynamic odor characteristics of CSC.

Electronic nose (E-nose) technology, inspired by the human olfactory system, integrates sensor arrays, signal-processing circuits, and pattern-recognition algorithms to detect and classify complex odors^11,12^. It offers advantages such as low power consumption, portability, and high sensitivity, and has been successfully applied in food safety^13^, air-quality monitoring^14^, and fossil-fuel analysis^15,16^. However, its potential for detecting spontaneous combustion in coal has received little attention. With the rapid advancement of miniaturized gas-sensing technologies, applying E-nose systems to CSC prediction has become not only feasible but also highly promising.

To interpret the complex response patterns of E-nose sensors, a robust pattern-recognition algorithm is required. Machine-learning techniques provide this capability by compensating for sensor limitations such as cross-sensitivity and mixed-odor interference^17–19^. They have demonstrated strong performance in modeling nonlinear, multidimensional data in CSC prediction tasks. For example, Onifade et al.^20,21^ developed artificial neural network (ANN) and metaheuristic-optimized ANN models that achieved high accuracy in predicting CSC liability indices. However, these models were primarily designed for gas-composition data rather than odor features. As a result, they fail to capture key properties of E-nose signals, including sensor cross-interference, redundancy, and nonlinearity, limiting their generalization ability when applied directly to CSC odor data.

In this study, a PTC E-nose testing system was developed to detect odor evolution during CSC. Feature extraction and analysis were conducted on the collected signals, followed by the construction of a machine-learning prediction model to classify CSC stages. By examining odor characteristics throughout the spontaneous combustion process, this study provides an experimental foundation for applying E-nose technology to early warning of coal fires. The proposed method aims to offer a rapid, cost-efficient, and automated approach for CSC monitoring in both mines and storage facilities. In particular, CSC tends to occur in goaf areas, fractured coal zones, fault zones, and abandoned roadways, where poor ventilation and residual coal accelerate oxidation and heating.

## Experimental design

### Experimental materials

The experimental coal samples were lignite with a low degree of coalification, collected from a mining face in Inner Mongolia. The coal samples were sealed and transported to the laboratory, where the surface oxidation layer was removed. The coal samples were then crushed and sieved to obtain particles sized 0.2–0.45 mm. Following the drying treatment, the samples were preserved, and programmed-temperature (PT) experiments were completed within 24 h. For each experiment, 50 g of the coal sample was used to ensure representativeness. The proximate and ultimate analyses of the coal sample are presented in Tables 1 and 2, respectively. M_ad_: Air-dried basis moisture content (mass fraction of moisture per unit mass of coal sample). A_d_: Dry basis ash content (mass fraction of inorganic residue after pyrolysis). V_daf_: Dry ash-free basis volatile matter (mass loss fraction after heating to a specific temperature under anaerobic conditions, excluding moisture effects). Ultimate analysis reports the mass fractions of elemental constituents. The study indicates that this coal sample has a high carbon content, low ash (A_d_) and moisture (M_ad_) content, and a high volatile matter (V_daf_) content. These characteristics contribute to its high calorific value and significant spontaneous combustion tendency. Based on the CSC propensity assessment report, the sample is classified as coal prone to spontaneous combustion.

**Table 1:** Proximate analysis results.

Proximate analysis	M_ad_/%	A_d_/%	V_daf_/%
Date	6.4	10.8	46.7

**Table 2:** Ultimate analysis results.

Ultimate	C	H	O	*N*	S
Quality fraction%	75.3	5.8	0.7	18.0	1.1

### Experimental apparatus and methods

#### Experimental apparatus

**Fig. 1:**
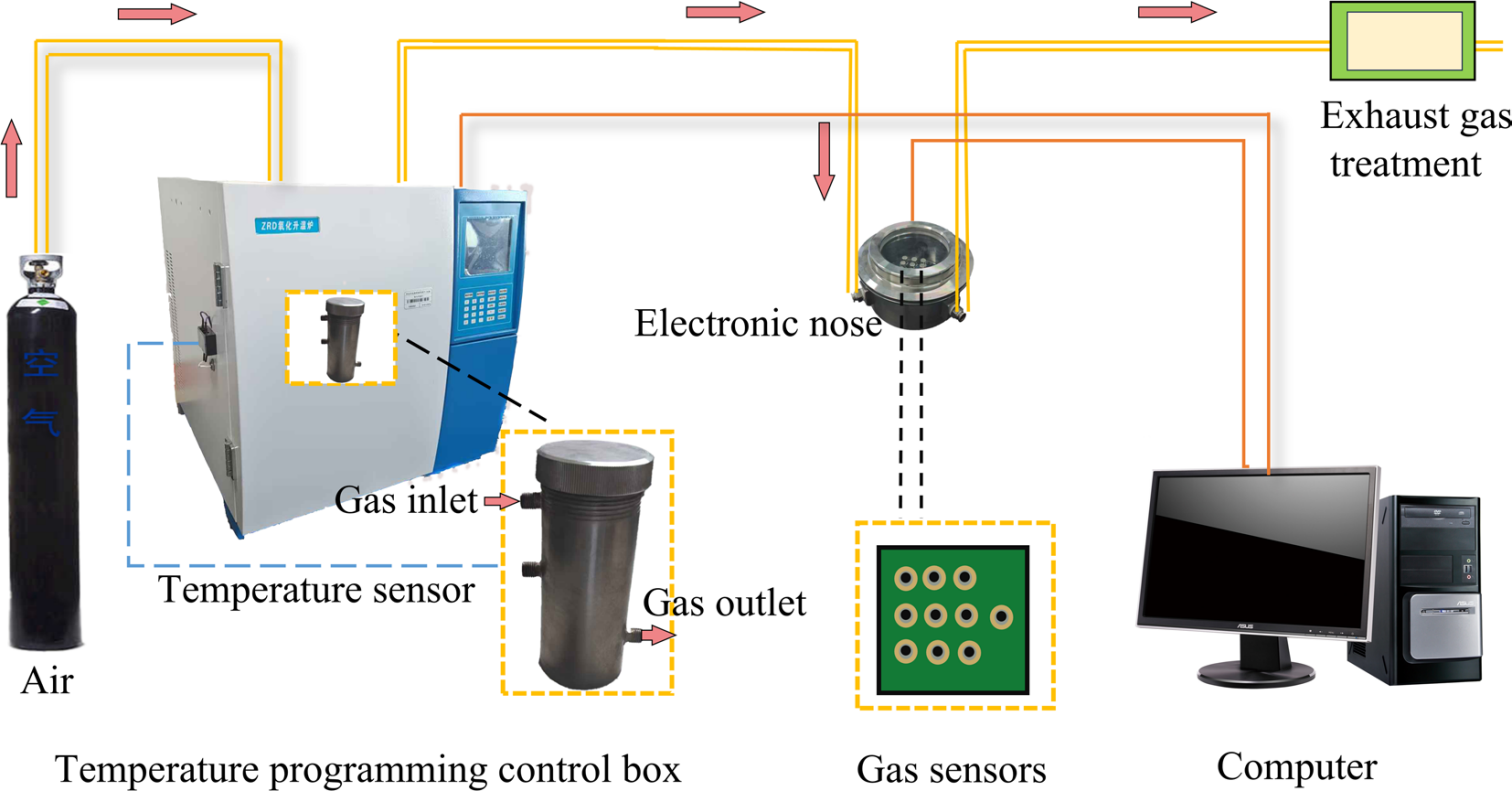
Programmed Temperature Coal E-Nose Testing Device.

The PTC E-Nose Testing Device is illustrated in Fig. 1. This apparatus consists of five main components: Gas Supply Module, comprising a high-purity air cylinder (providing carrier gas), flow control valves, and a silica gel desiccation tube. This module supplies and conditions the carrier gas flow entering the coal sample. Temperature Control Module: Integrated with the coal sample furnace to form the core PT device. Its central component is a copper-coal sample chamber equipped with internal temperature sensors; it is externally wrapped in thermal insulation and heating materials for precise PT control. The Sampling Module includes a flow meter and gas sampling bags. It is designed for the quantitative collection and storage of gaseous products from the coal sample furnace. This module is integrated with the temperature control module.

**Table 3:** Properties of gas sensors.

Serial number	Sensor model	Detection object	Sensitivity
a	C1	Ethanol, Natural gas	1-100ppm
b	C3	Benzene, Toluene	0.1-50ppm
c	C4	Ketones	1-200ppm
d	D1	Methane, Butane, Propane	1-5000ppm
e	D2	Ammonia	1-30ppm
f	D3	VOC, Alcohol	10-1000ppm
g	D4	Nitrogen dioxide	0.01-20ppm
h	D5	Sulfur dioxide	0.1-1000ppm
i	D6	Hydrogen sulfide	1-100ppm
j	Mos-1	Acetaldehyde	0.1-500ppm

The E-nose system comprises three functional components: the gas-sensor-array detection module (hereafter “detection module”), the test chamber, and the test-analysis software. The detection module, responsible for sensing compositional variations in the sampled gas, incorporates a multi-channel header plate with ten gas-sensitive sensors mounted; their specifications are listed in Table 3. These sensors were selected based on their specific sensitivities to the volatile gas components characteristic of mine fires, as established in prior research^9,10^. Additionally, the sensor array was kept concise, and the test chamber volume was minimized to ensure that each sensor detects stable odor signals. As a result, the types and quantities of gas sensors were determined based on these two design considerations. The response of each sensor was characterized by its differential signal between exposure to CSC gases and baseline in clean air. The test chamber provides a stable environment for the sensors; it is a stainless-steel cylindrical vessel with a volume of 400 ml. The test analysis software acquires and processes, in real time, the signals generated by the detection module via the data collection interface.

#### Experimental procedure

To investigate how the odor signal of CSC gases evolves with temperature, a PT Coal method was used to collect odor signatures from the coal sample at discrete temperatures. The detailed procedure is as follows:

1) Connect each module of the experimental apparatus as illustrated in Fig. 1. Power on the PT device and the E-nose system for preheating. Inspect the entire setup to confirm that it is hermetically sealed.

2) Configure the PT parameters with a carrier-gas flow rate of 100 mL/min, a heating rate of 0.5 °C/min, a temperature range of 30–200 °C, and gas sample collection every 5 min.

3) Set the e-nose parameters to activate the sensor heater at 1.8 V with a sampling frequency of 1 Hz.

4) Heat the coal sample to 30 °C and maintain this temperature for 30 min to ensure thermal equilibrium, then initiate the PT process.

5) Collect gas samples in polyethylene gas bags and store them at a constant temperature after each sampling. Terminate the temperature program after collecting the gas sample at 200 °C.

6)Introduce the collected gas samples into the e-nose test chamber using a gas-tight syringe at 60 mL per injection. Based on prior single-factor optimization, we established the following test conditions: a sensor baseline stabilization time of 50 s, a data-acquisition window of 120 s, and sensor purge and recovery times of 90 s.

**Fig. 2:**
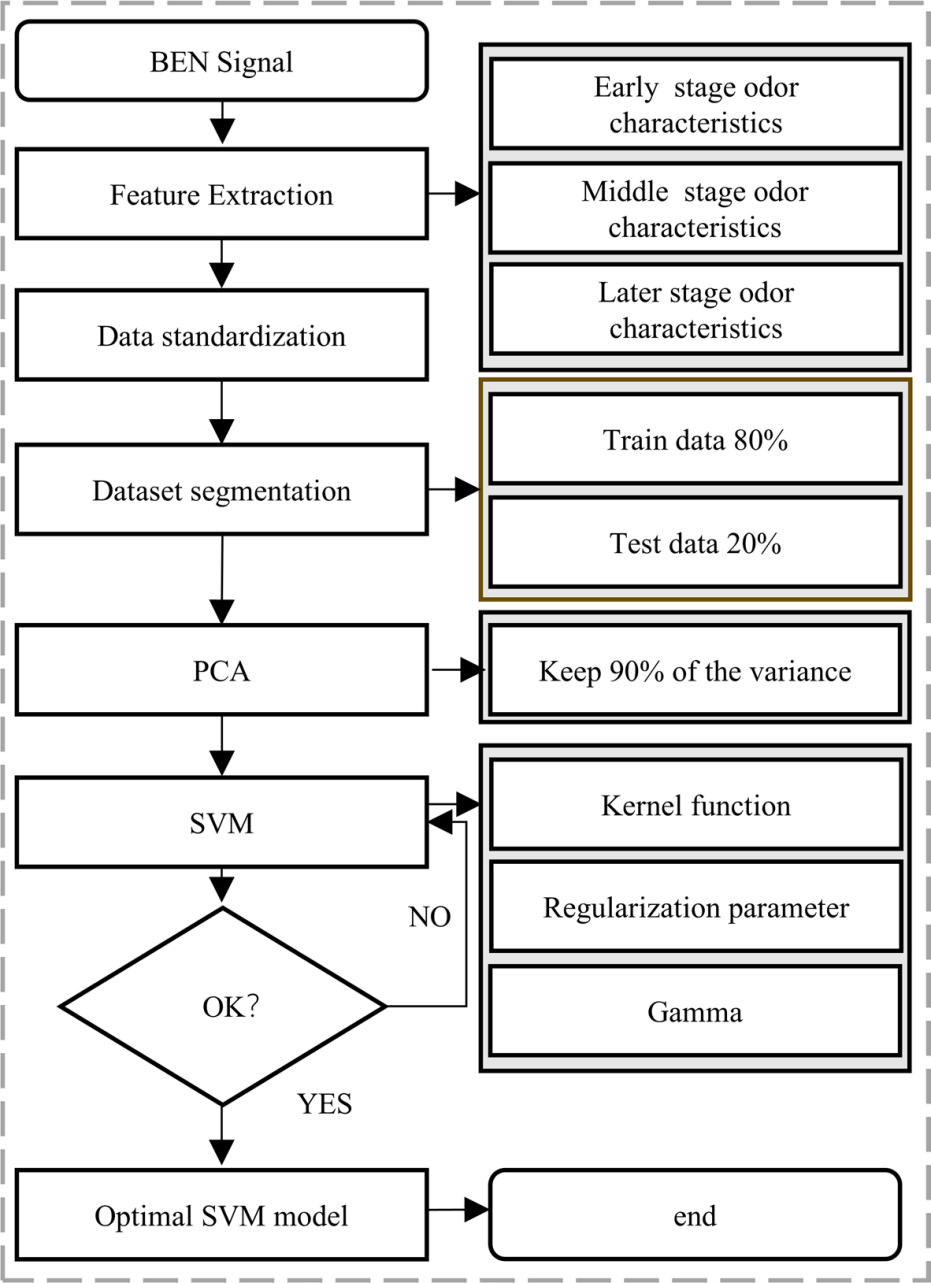
Establishment Process of PCA-SVM Model.

### Analytical methods

#### Feature analysis

The raw response signals (characteristic signals) acquired by the E-nose system for CSC gases at different temperatures cannot directly reflect the relationship between odor characteristics and temperature variation. In this study, the random forest algorithm’s feature-importance metric was employed to identify and select sensors whose responses exhibit the strongest correlation with temperature-dependent odor evolution. Polynomial and Gaussian curve-fitting methods were then applied to examine how the response intensities of these key sensors vary with temperature throughout the CSC process. This analysis aims to verify whether the response intensities of the selected sensors are suitable as practical input features for a model predicting CSC.

#### Principal component analysis (PCA)

PCA is a widely employed multivariate technique in E-nose data processing. After feature extraction from the raw sensor responses, the dataset still comprises ten sensor channels (10-D). PCA performs an orthogonal linear transformation that projects the original features onto a new coordinate system. This projection suppresses noise while preserving the dominant information.

In this study, PCA was applied to the 10-D feature matrix obtained from the E-nose. The resulting principal components were ranked by explained variance, and their cumulative contribution was evaluated. By inspecting the sample coordinates in principal component space, 2-D and 3-D score plots were generated. These visualizations reveal the distribution patterns and clustering of odor signatures across different stages of CSC. This result substantiates the rationality of classifying these stages based on olfactory data.

#### CSC odor prediction model

CSC Odor Prediction Model: An integrated modeling approach combining PCA and SVM was adopted to construct a prediction model for the stages of CSC. The detailed workflow is illustrated in Fig. 2 and comprises the following steps:

1) Data Preparation and Partitioning: According to the developmental characteristics of CSC, samples were labeled as early, middle, or late stage. We randomly split the dataset into a training set and an independent test set at an 8:2 ratio.

2) Feature Dimensionality Reduction: PCA was applied to the training features to reduce dimensionality and remove noise. Principal components whose cumulative variance contribution exceeded 90% were retained, yielding the reduced feature set for training.

3) Model Training and Comparison: Using the PCA-reduced training features as input, an SVM model was trained to establish the PCA–SVM classifier. Additionally, standalone SVM, Artificial Neural Network (ANN), and Random Forest (RF) models were trained. These models serve as the paradigm for classic odor classification and as a reference for new models. Their overall statistical performance metrics were analyzed to verify the PCA–SVM model’s superiority for classifying CSC stages.

4) Each model was subsequently evaluated by means of a confusion matrix and four assessment metrics, namely Accuracy, Recall, Precision, and F1-score (denoted as f1_score). The respective formulae are presented below:

1 $$Accuracy=\frac{{TP+TN}}{{TP+TN+FP+FN}}$$

2 $$Recall=\frac{{TP}}{{TP+FN}}$$

3 $$Precision=\frac{{TP}}{{TP+FP}}$$

4 $$FI\_score=2 \times \frac{{\left( {Precision \times Recall} \right)}}{{\left( {Precision+Recall} \right)}}$$

Here, we define TP as true positive, FN as false negative, FP as false positive, and TN as true negative to evaluate the model’s performance.

5) For the selected optimal model, stratified k-fold cross-validation was employed to evaluate its performance. By repeatedly partitioning the data, the consistency and generalization capability of the optimal model were rigorously assessed.

All experiments were conducted in Python 3.8, primarily using the scikit-learn machine learning library.

## Results and discussion

### Odor characteristics of CSC

**Fig. 3:**
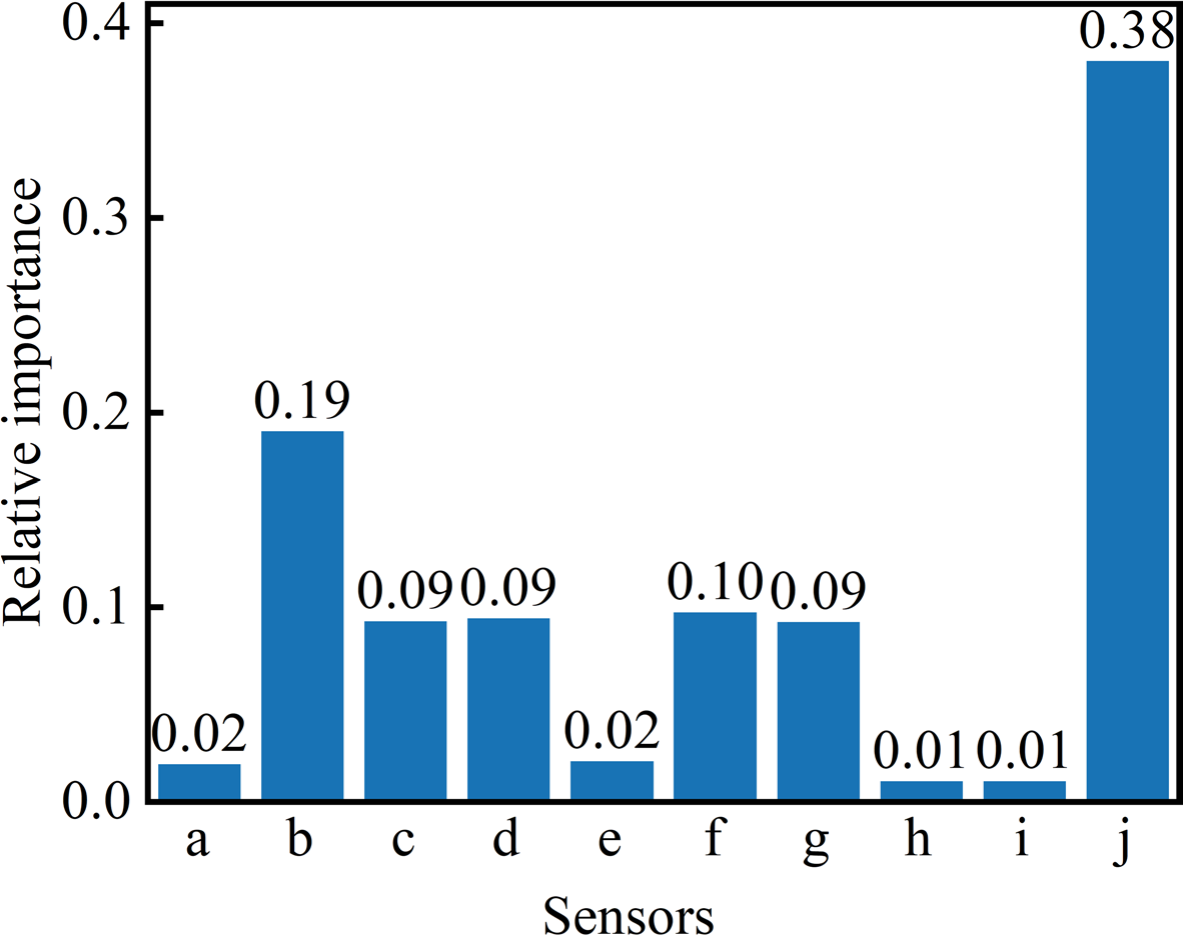
Sensors’ importance: CSC stage by random forest as a classification model. The abscissa denotes the sensor array index; the ordinate represents the feature importance (summing to 1).

Using the baseline-difference method, response intensities of the ten sensors to CSC gases at different temperatures were calculated. Combined with the random forest model’s feature-importance assessment, the importance scores for the individual sensors were obtained, as shown in Fig. 3.

Figure 3 indicates that array j (MOS-1 sensor, acetaldehyde-sensitive) and array b (benzene-type sensor) exhibit the highest feature-importance values—0.38 and 0.19, respectively—demonstrating that these two sensors capture the dominant odor signatures of CSC. Sensors c, d, f, and g show feature-importance scores between 0.05 and 0.08, implying minor contributions from ketone, alkane, amine, and VOC emissions during the process. Arrays a, e, h, and i yield importance scores below 0.02, reflecting the weak odor signals detected by these channels under the experimental conditions.

**Fig. 4:**
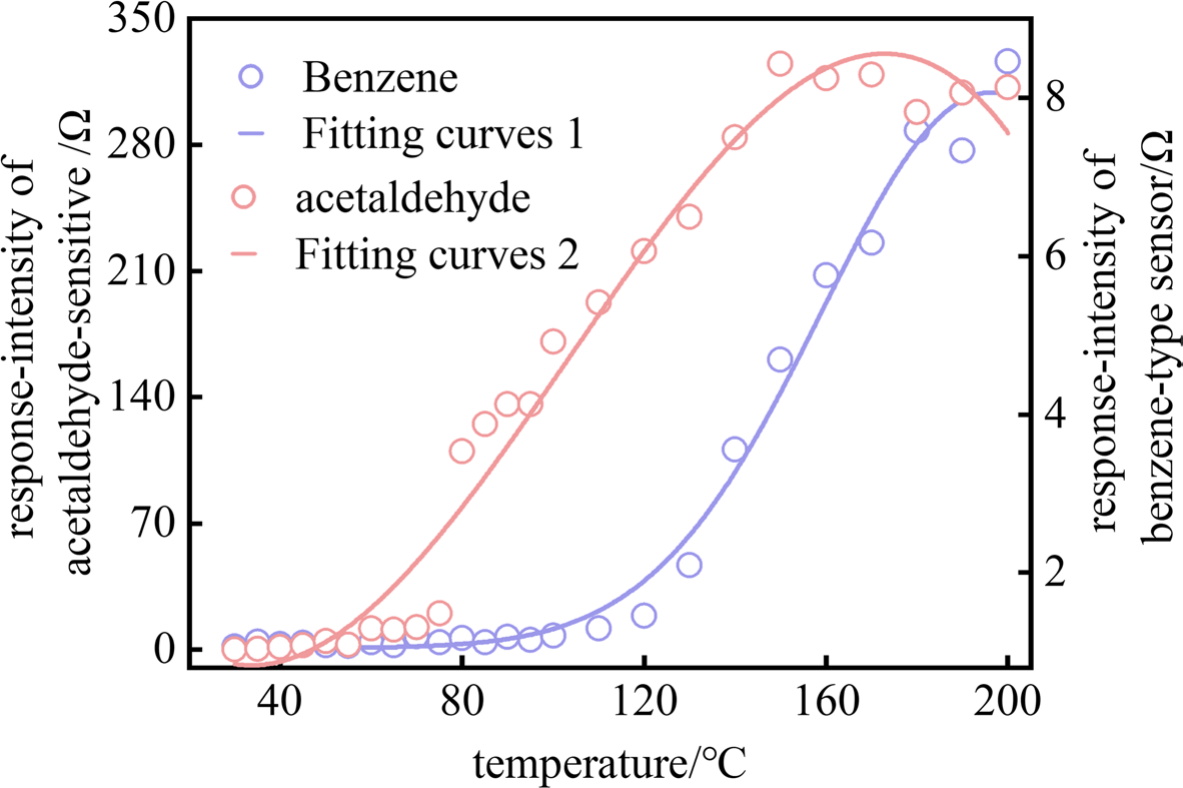
Variation of response intensity and its fitting curve for acetaldehyde and benzene sensors.

The response-intensity curves for the acetaldehyde (array j) and benzene-type (array b) sensors, along with their fitting results, are presented in Fig. 4. As shown, acetaldehyde is first detected at 60 °C; its response intensity rises steadily with temperature, peaks at 160 °C, and then stabilizes with minor fluctuations. A polynomial fit reveals a strong positive correlation between response intensity and temperature (R² = 0.98), confirming acetaldehyde as an effective indicator for early-stage CSC. The fitting function is:

5 $$F({x_1})=0.0003{x_1}^{3}+0.078{x_1}^{2} - 4.3905{x_1}+60.4157$$

In the acetaldehyde fitting equation, x₁ denotes the independent variable (temperature), and F(x₁) denotes the dependent variable (predicted sensor response intensity at temperature x). Benzene-type compounds exhibit no significant response below 120 °C; above this threshold, the intensity rises continuously with temperature. A Gaussian fit yields a high correlation (R² = 0.98), with the fitting function:

6 $$F({x_2})=7.018 \cdot \exp \left( {\frac{{ - {{\left( {{x_2} - 196.131} \right)}^2}}}{{2 \cdot {{\left( {37.046} \right)}^2}}}} \right)+1$$

In the benzene-class fitting curve, x₂ is the independent variable representing temperature, F(x₂) is the dependent variable representing the fitted sensor response intensity at temperature x. This makes benzene-type emissions a valuable supplementary marker for the late stage of CSC. The odor-characteristic analysis of CSC reveals distinct stage-dependent features. This is consistent with the research findings of Refs^9,10^.

### Stage division of CSC

**Fig. 5:**
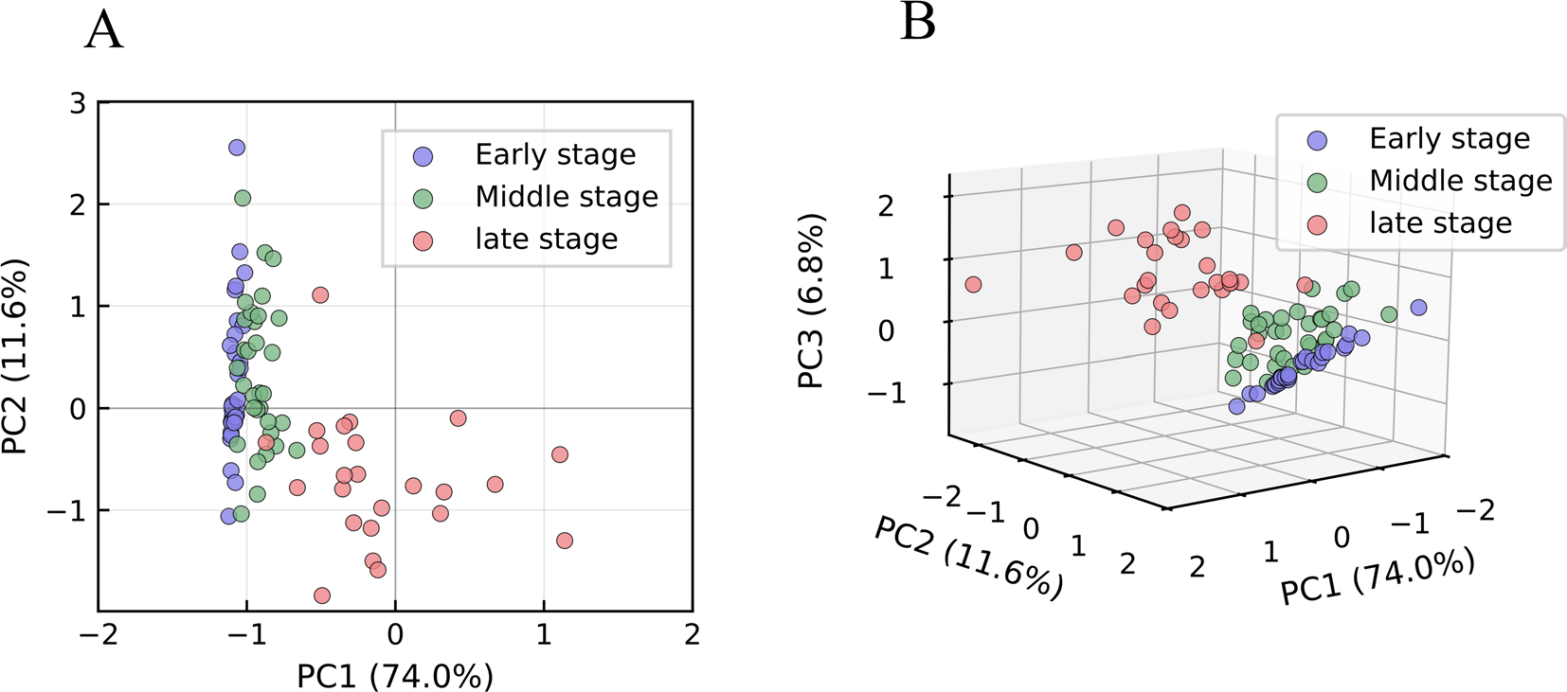
PCA 2D and 3D score plot illustrating the distribution of odor features during the early, middle, and late stages of CSC.

Based on the temperature-dependent evolution of odor characteristics, the spontaneous-combustion process was divided into three stages: early (30–60 °C), middle (60–130 °C), and late (> 130 °C). PCA was performed on odor signatures from these three stages. Figure 5 presents the 2-D and 3-D PCA score plots for the different stages.

In the 2-D plot, the first and second principal components account for 74.3% and 11.6% of the variance, respectively; in the 3-D plot, the third principal component accounts for an additional 6.8%. Together, the first two components explain 85.6% of the total variance, and the first three components explain 92.4%—both exceeding the 85% threshold.

These results indicate that the first three principal components adequately capture the discriminatory information contained in the E-nose response signals. Although there is a minor overlap between the early- and middle-stage samples, the clusters remain distinguishable. The late-stage samples are clearly separated from the early and middle samples. This separation arises because oxidation proceeds relatively gently during the early and middle stages, producing fewer odor species at lower concentrations. In contrast, the late stage is characterized by vigorous oxidation that generates abundant odor compounds at elevated concentrations. Both the 2-D and 3-D PCA plots successfully differentiate the early, middle, and late stages, demonstrating that E-nose odor data can serve as a reliable basis for CSC stage classification.

### CSC odor recognition model

#### Model training

Both the temperature-dependent odor characteristic analysis of CSC and PCA demonstrate that odor variation can be used to demarcate its stages. PCA indicates that the first three principal components account for more than 90% of the variance; in conjunction with a support vector machine (SVM), these components were used to establish a PCA–SVM odor prediction model for CSC. The optimal SVM parameters are: kernel = linear; C = 100; gamma = 0.01.

#### Model identification experiment

Following the same rationale used to construct the PCA-SVM model, this study also established standalone SVM, RF, and ANN classifiers for stage discrimination, thereby benchmarking the PCA-SVM approach. The optimal hyperparameters for each model are listed in Tables 4 and 5.

**Table 4:** Performance parameters of the PCA-SVM model and the SVM model to predict CSC. In the Table, ES, MS, and LS refer to the early, middle, and late stages of CSC.

Parameters	PCA-SVM			SVM		
	ES	MS	LS	ES	MS	LS
f1_scorem	1.00	1.00	1.00	0.92	0.80	0.83
Recall	0.86	0.86	0.86	0.86	1.00	0.71
precision	0.83	0.92	0.92	1.00	0.67	1.00
Accuracy	0.95			0.85		

**Table 5:** Performance parameters of the RF model and the ANN model to predict CSC.

Parameters	RF			ANN		
	ES	MS	LS	ES	MS	LS
f1_scorem	1.00	0.67	0.71	0.92	0.86	0.92
Recall	1.00	0.67	0.67	0.86	1.00	0.86
precision	1.00	0.71	0.67	1.00	0.75	1.00
Accuracy	0.80			0.90		

**Fig. 6:**
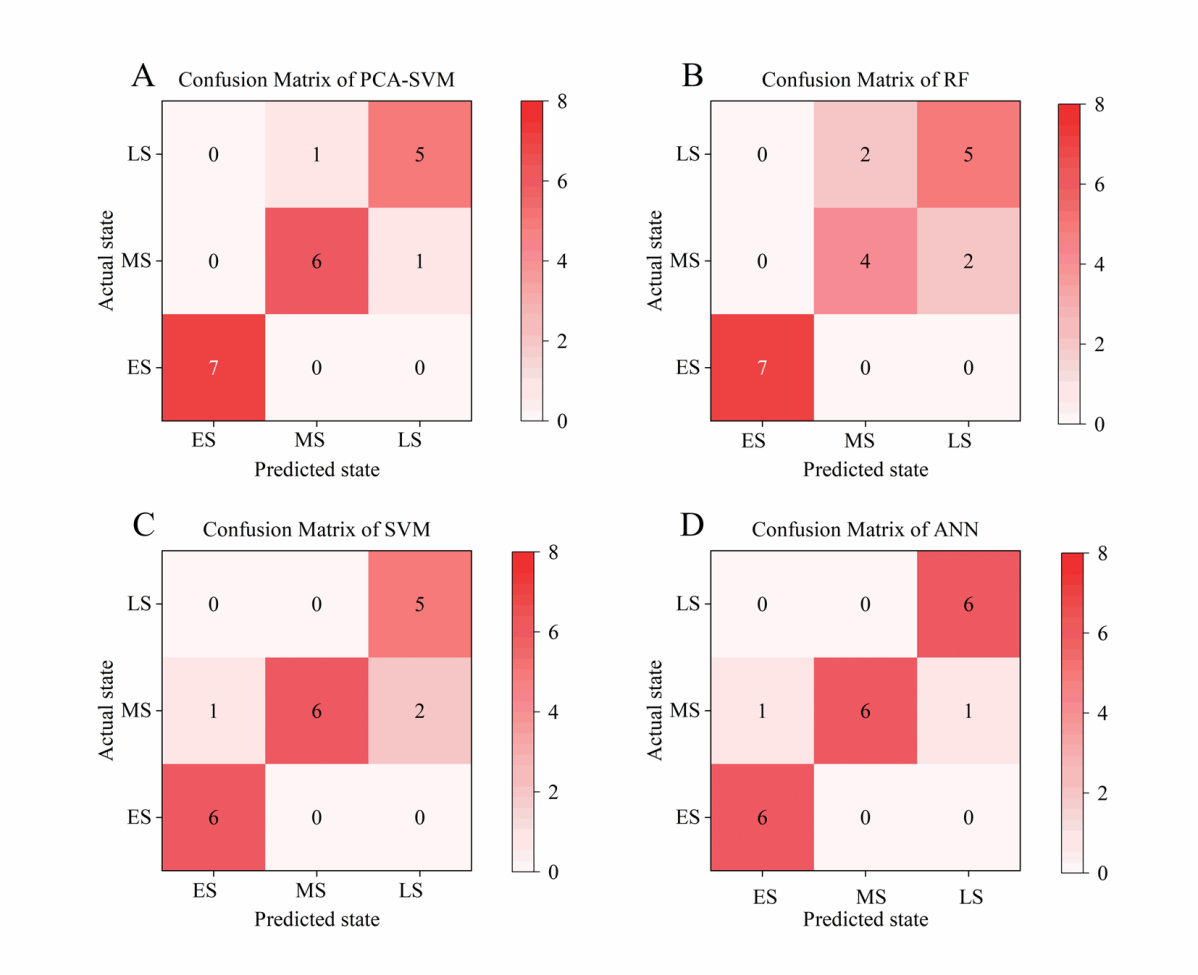
Confusion matrix of the **(A)** PCA-SVM model, **(B)** RF model, **(C)** SVM model, **(D)** ANN model. In the image, ES, MS, and LS refer to the early, middle, and late stages of CSC.

As indicated in Tables 4 and 5, and Fig. 6, all models achieved accuracies above 85%. The PCA-SVM model achieved the highest accuracy of 95%, whereas the alternative models achieved accuracies of 85% for RF, 90% for ANN, and 89% for standalone SVM. A detailed analysis shows that the PCA-SVM correctly identified 100% of early-stage samples. For the middle and late stages, accuracy was slightly lower: one middle-stage sample was misclassified as late, and one late-stage sample as middle. RF exhibited the lowest overall accuracy; its precision at the middle stage was only 57.1%. ANN achieved 90% accuracy but showed signs of overfitting, suggesting limited generalization to unseen data. Standalone SVM achieved 89% precision; after PCA-based dimensionality reduction, accuracy rose to 95%. This improvement demonstrates that the raw sensor signals contain redundant features or noise, and that PCA effectively enhances model precision by removing them.

#### Model validation

Stratified five-fold cross-validation was performed on the PCA–SVM model. The resulting fold-wise accuracies were 0.80, 0.92, 0.85, 0.86, and 0.87, respectively. Except for the first fold (0.80), all other folds exceed 0.85, yielding an average accuracy of 0.86 (> 0.85). Accuracy, recall, and F1-score all remain above 0.85, confirming the model’s stability and generalization capability.

The PCA–SVM model outperformed the other classifiers in identification accuracy, with lower mean-squared and mean-absolute errors. Therefore, the PCA–SVM model demonstrates superior performance for monitoring and identifying the CSC stage. This corroborates the finding that PCA improved the SVM model’s performance in recognizing CSC odors.

Compared with conventional methods^4–8^, the E-nose offers a compact and integrated solution for CSC monitoring. Its portability and built-in intelligence allow real-time, dense monitoring in mines, significantly improving early warning and operational safety.

## Conclusions

This study investigated the feasibility of predicting CSC stages using an E-nose system combined with machine learning. The results showed that CSC emits characteristic VOCs, primarily aldehydes, benzene derivatives, and ketones, whose concentrations vary significantly across combustion stages. Random-forest feature ranking revealed that the acetaldehyde and benzene sensors contributed most to stage discrimination, with importance scores of 0.38 and 0.19, respectively. PCA indicated that the first three components explained 92.4% of the total variance, effectively capturing the key odor features of CSC. Based on these features, a PCA–SVM classification model was developed to identify the early, middle, and late stages of CSC, achieving 95% prediction accuracy. The model exhibited strong stability and generalization capability under cross-validation, confirming that E-nose odor data can serve as a reliable basis for CSC monitoring.

These results demonstrate that integrating E-nose technology with machine-learning algorithms provides a rapid, low-cost, and automated approach for early warning of CSC in mining and storage operations. The system also has potential for application in high-risk regions such as goaf areas, fractured coal zones, fault zones, and abandoned roadways.

Despite the promising results, several challenges remain before practical field deployment. (1) Environmental adaptability: The current experiments were conducted under laboratory conditions; dust, humidity, and airflow in real mine environments may affect detection accuracy. Future work should develop protective sampling modules and adaptive signal-compensation mechanisms. (2) Coal diversity: The study focused on lignite samples. Further investigation of coals with varying ranks and metamorphic grades is required to improve model generalization.

## Data Availability

The datasets used and/or analyzed during the current study are available from the corresponding author on reasonable request.
